# SARS-CoV2 (COVID-19) Pandemic Lockdown Influences Nature-Based Recreational Activity: The Case of Birders

**DOI:** 10.3390/ijerph17197310

**Published:** 2020-10-07

**Authors:** Christoph Randler, Piotr Tryjanowski, Jukka Jokimäki, Marja-Liisa Kaisanlahti-Jokimäki, Naomi Staller

**Affiliations:** 1 Department of Biology, Eberhard Karls University Tuebingen, Auf der Morgenstelle 24, D-72076 Tuebingen, Germany; naomi.staller@uni-tuebingen.de; 2 Institute of Zoology, Poznan University of Life Science, Wojska Polskiego 71C, 60-625 Poznań, Poland; piotr.tryjanowski@gmail.com; 3 Arctic Centre, University of Lapland, P.O. Box 122, FI-96101 Rovaniemi, Finland; jukka.jokimaki@ulapland.fi (J.J.); marja-liisa.kaisanlahti@ulapland.fi (M.-L.K.-J.)

**Keywords:** birding, birdwatching, citizen science, recreation, leisure, behavioral changes

## Abstract

The new corona virus infection SARS-CoV2 which was later renamed COVID-19 is a pandemic affecting public health. The fear and the constraints imposed to control the pandemic may correspondingly influence leisure activities, such as birding, which is the practice of observing birds based on visual and acoustic cues. Birders are people who carry out birding observations around the globe and contribute to the massive data collection in citizen science projects. Contrasting to earlier COVID-19 studies, which have concentrated on clinical, pathological, and virological topics, this study focused on the behavioral changes of birders. A total of 4484 questionnaire survey responses from 97 countries were received. The questionnaire had an open-ended style. About 85% of respondents reported that COVID-19 has changed their birding behavior. The most significant change in birdwatchers’ behavior was related to the geographic coverage of birding activities, which became more local. People focused mostly on yard birding. In total, 12% of respondents (*n* = 542 cases) reported having more time for birding, whereas 8% (*n* = 356 cases) reported having less time for birding. Social interactions decreased since respondents, especially older people, changed their birding behavior toward birding alone or with their spouse. Women reported more often than men that they changed to birding alone or with their spouse, and women also reported more often about canceled fieldtrips or society meetings. Respondents from higher developed countries reported that they spend currently more time for birding, especially for birding alone or with their spouse, and birding at local hotspots. Our study suggests that long lockdowns with strict regulations may severely impact on leisure activities. In addition, a temporal and spatial shift in birding due to the pandemic may influence data quality in citizen science projects. As nature-based recreation will be directed more toward nearby sites, environmental management resources and actions need to be directed to sites that are located near the users, e.g., in urban and suburban areas. The results can be applied with caution to other nature-based recreational activities.

## 1. Introduction

The SARS-CoV2 (COVID-19; hereafter) infection is the first pandemic in times of modern technological societies with such strong governmental precautionary measures. The pandemic has severe impacts on health and well-being, with a total of 11,470,637 global infection cases and 534,784 deaths in 188 countries on 6.7.2020 [1]. On the one hand, direct lethal outcomes have an impact on life, but on the other, governmental restrictions to keep the virus spreading under control also affected nearly all aspects of life and public health, e.g., daily traffic [2], or psychological and social aspects [3,4]. The importance of social and psychological factors for mental and physical well-being is well known, and one aspect is the effect of leisure activities on physical and mental health [5]. In this study, we used birders as an example for a non-consumptive nature-based outdoor leisure activity. Based on our knowledge, this is the first study assessing the lockdown impact on birders. In addition, it is one of few studying the COVID-19 pandemic impact on a leisure activity on a global scale by asking individuals about their birding behavior during the pandemic. Our study suggests that long lockdowns with strict regulations may severely impact leisure activities. The results can be applied with caution across other nature-based recreational activities.

## 2. Theoretical Background

### 2.1. Recreation Specialization

Recreation specialization is concerned with a leisure activity that needs some affective, motivational, and cognitive effort [6,7]. Recreation specialization represents a continuum in behavior between the generalists with a low involvement and the specialists with a high involvement [6]; meaning that some people invest more time, effort, and cognitive resources than others. Scott and Shafer [7] defined a three-dimensional construct with a behavioral, a cognitive, and an affective component. Indicators of the behavioral component are equipment, previous participation, and experience. Indicators of the cognitive dimension include the level of competence and knowledge of the activity. Indicators of the affective component include lifestyle centrality and continued participation [7,8]. Centrality to lifestyle has a psychological and a behavioral component [8] and can be viewed as part of the larger construct of involvement [9]. Social-psychological involvement is a state of motivation, arousal, or interest regarding a product, an activity, or an object [10]. Birders can be referred to as experts in the field of identifying bird species. However, their skills may differ, e.g., between men and women (see Section 2.2 and Section 2.3 for more details). 

### 2.2. Birding as Leisure Activity

Birding is an important nature-based recreation activity. For example, about 18% of Americans watch birds and about 36% of them carry out birding trips away from their homes [11,12]. The concept of recreation specialization has also been adapted to birders. McFarlane [13] used the factors “past experience”, “economic commitment”, and “centrality to lifestyle” to group birders into four categories: novice, casual, intermediate, and advanced birders. Hvenegaard [8] categorized birders into three distinct groups: advanced-experienced, advanced-active, and novices. Scott and Thigpen [14] identified four groups of birders, namely casual, interested, active, and finally, skilled birders. More colloquial definitions sometimes group people into birdwatchers, birders, and twitchers [15,16]. A special case has been described in birders that travel long distances to see rarities [17]. This behavioral commitment defines them as hard-core birders. Birding follows the dimensions of recreational specialization [6], including aspects of skill and knowledge, behavioral and personal commitment, stages of involvement [10], and centrality to lifestyle [9]. A recent study has highlighted that there is a significant positive relationship between recreational specialization and birder’s travel intention [18]. Additionally, demographic factors may influence the specialization level of birders (see Section 2.3. for more detailed explanation). Therefore, birding is an ideal nature-based recreation activity to study the influence of the COVID-19 lockdown on a common leisure and recreational activity, because it requires activity mostly outside in nature, travelling to birding areas, and it often includes a social component, such as meeting other birders. Voluntary birders (experienced citizens as well as laypersons) also play an important role on large-scale biodiversity surveys and bird population monitoring. Birders around the globe contribute to the massive data collection that is used for scientific studies [19,20]. The effort of leisure birding to ornithological science is unparalleled and allows studies (e.g., of bird migration patterns) that could not be conducted by paid professionals on a large scale [20]. The knowledge about changes in birding behavior is important because it may have strong consequences for the data collection and long-term analyses of bird data. For example, a recently published study from South Africa stated that the COVID-19 governmental measures had a marked negative effect on the data collection for the Southern African Bird Atlas Project. Due to restricted mobility, there was a 70% decline in data reporting in April 2020 [21]. Since there is no previous comparable situation, this part of our research is explorative.

### 2.3. Demographic Factors: Gender and Age 

Birding data suggests that men usually display a higher level of specialization. For example, male members of the Carolina Birding Club (USA) reported higher skills and more expensive birding equipment [22]. Scott and Thigpen [14] found that men participated more frequently in birding activity, travelled longer distances, and reported higher identification skills than women. However, there was no difference between genders in level of commitment. Men are also more competitive in their birding activities [15].

Other well-known psychological differences between men and women can contribute to gender-specific behavioral responses during the COVID-19 outbreak. First, men score significantly higher in risk-taking than women in many tasks as found in the meta-analysis by Byrnes et al. [23]. Similarly, men tend to rule-breaking behavior and delinquency more than women [24]. Further, women were more anxious than men in the general trait anxiety or neuroticism [25]. In terms of personality dimensions, women scored higher than men in neuroticism-anxiety, while men scored higher in sensation seeking [26]. Combining this evidence, we predict that women experience more restriction in their birding behavior, because they may be more anxious about the disease, try to reduce risk of infection, and stick to governmental rules more than men.

In general, life experience comes with age, and experienced people are predicted to take fewer risks. From a personality psychology viewpoint, age is related to a higher self-control, emotional and mood stability, and especially to lower sensation seeking [27]. In addition, many countries set movement restrictions and quarantines for older people and different risk groups because COVID-19 seems to affect those more strongly [28].

### 2.4. Impact of the COVID-19 Pandemic on Human Behavior

During the COVID-19 pandemic, many countries adopted restrictive measures based on physical (“social”) distancing, to prevent human-to-human virus transmission [29]. This was termed “lockdown”. Lockdown is a colloquial term for “mass quarantine”, based on “stay-at home” or “shelter-in-place” orders [29]. Behavioral changes, especially, reducing large recreational gatherings, which are considered superspreading events, were one option to reduce the virus spreading [30]. However, quarantine and prolonged home stay may have severe side effects, such as physical inactivity and social isolation [29]. Activity in nature seems to be a protective factor for public health [31]. For example, Pasanen et al. [32] showed that emotional well-being was positively related to physical activity in nature. Still, governmental and organizational information can guide people to avoid infections (e.g., keeping physical distance, wearing masks). Despite the fact that short-time quarantine can slow down or restrict the spread of COVID-19, long-time isolations can cause both economical and mental problems.

Currently, global, national, regional, or even local restrictions for travelling have been one of the main actions to prevent the spread of COVID-19 [30]. Additionally, even adjacent countries employed different strategies, such as Finland with strong restrictions and Sweden with only less and mild restrictions. This may have consequences, e.g., on nature-based tourism and recreation activities across multiple spatial scales. For example, at the national level, many countries restricted entry for foreign tourists [33]; and local people were unable to use bird towers and nature centers for their recreation because these sites were closed [34]. 

People could also change their daily or circadian area use due to COVID-19. For example, they may avoid, e.g., shopping during the rush hours. Many shops have arranged special visiting hours with few customers for elderly and other risk groups. Temporal changes were found in nature-based recreation. For example, in Norway, outdoor recreational activity increased by 291% during lockdown relative to a 3-year average for the same days [35]. Pedestrian activity increased in city parks, peri-urban forest, as well as protected areas [35]. Additionally, many companies started remote working or shortened working times, which may lead to more time at home and more time for birding.

### 2.5. Hypotheses and Predictions

In the context of the pandemic lockdown, we have five main hypotheses on the behavior of birders, based on the theoretical background outlined above.

**Hypothesis** **1.** *COVID-19 influences where birders hold their activities.*

Thus, it will influence the spatial area use of birders because of workplace closings, stay at home requirements, as well as international and domestic travel restrictions [2,33]. Many birders travel, sometimes also outside from their home country, to interesting birding sites, and to see rare bird species [14,15,22,36]. Therefore, we predict that birders should travel less, do less long-distance birding trips, and observe birds closer to their homes or even focus on their yards during the pandemic.

**Hypothesis** **2.** *COVID-19 influences when birders hold their activities.*

Thus, it will change the temporality of birding. In general, people may have more time for leisure, like birding, because they are forced into remote working and/or partial unemployment. We also hypothesized that birders will try to avoid rush hours in preferred birding sites, like bird towers. Basically, birding can be done throughout the day and on a daily basis, but most birders go to watch birds during the weekends when they have more time, and when the birds are most active, i.e., just before or during sunrise [11,37].

**Hypothesis** **3.** *COVID-19 influences birders’ social behaviors.*

As birding is to some extent a social activity [8,13], we expect that birders should complain about a decrease in group-birding with their friends (other than their spouse or partner) due to restrictions on gatherings and social (physical) distancing rules [29,30]. We also predict that birders will report cancelations of common birding events that decrease their sociality.

**Hypothesis** **4.** *The gender and age of birders influence their behaviors in the COVID-19 context.*

We predict that older birders change their behavior more than younger ones, because younger people are predicted to take more risks than older people [28] and may stick less to governmental restrictions [27]. We also predict that women will change their birding behavior more than men, because men differ from women in the behavioral component in birding and in competitiveness [8,18,22]. Men are also more risk-taking than women; therefore, men might ignore the restrictions more easily [23,24,25,26].

**Hypothesis** **5.** *Country of residence influences birders’ behavior in the COVID-19 context.*

Most countries have reacted heavily to the COVID-19 crisis, e.g., by engaging in social distancing and different kinds of closures [38]. Consequently, international tourist arrivals (overnight visitors) have decreased over a half from January to May 2020 compared to the same period in 2019 [39]. Additionally, birders travel a lot, at least those that take birding seriously, e.g., twitchers. However, countries differ widely in their resources to detect, prevent, and respond to outbreaks [40,41]. We hypothesized that there will be differences in birders’ behavior between countries due to COVID-19. We predict that birders living in higher developed countries (with high human development index value; see Section 3.3. for more details) and individuals having more resources (money) to travel) will be more affected by the COVID-related factors (like travelling restrictions) than birders living in developing countries (with a low HDI value) [8,14]. Because countries have different strategies against the COVID-19 pandemic, we predict that birders living in countries with a strong containment and closure policy (a high stringency index value; see Section 3.3 for more details) will be more affected than birders living in countries with a slight policy (a low stringency index).

## 3. Material and Methods

Most birders are males [13] and about 50 years of age [14]. Our sample fits well to the age and gender bias of previous work. Thus, we consider the sample as representative. Birding activity culminates in northern temperate latitudes in spring, especially when songbirds start singing and migrant birds arrive back from their winter quarters in the southern hemisphere [42]. This period matched with the spread of COVID-19 to those areas. 

### 3.1. Data Collection

We started our global questionnaire survey on 30 March 2020 and continued until 2 May 2020. Therefore, we collected data during the most restrictive lockdown measures in most of the countries. A recruitment e-mail was sent over Facebook, Twitter, mailing lists, social media, and websites (see Supplementary Material File S1). The recruitment email letter and the questionnaire were available in English, Arabic, Farsi, Finnish, French, German, Greek, Polish, Portuguese, Spanish, and Italian. We asked to distribute the questionnaire widely. We also contacted 176 birding organizations, websites, or magazines and asked them to publish the recruitment letter and the link to the questionnaire in their online systems like newsletters, Facebook groups or announcements. Eighty-one “closed” Facebook groups were joined and a bird photo was posted with the announcement to support the study (Supplementary Material File S2a,b show two postings within organizations; Supplementary Material File S3 shows a posting on Facebook). About 2000 e-mails were sent during the recruitment. The recruitment mail was sent to friends and colleagues in many countries (≈200), birding e-mail listserv (85: a total of 75 helped us), and to other birders (about additional 1500 e-mails).

The questionnaire was hosted on the German SoSciSurvey server to fulfil the European Union’s data privacy rules. The questionnaire asked one open-ended question: “Did your birding activity change during the Corona crisis? If so, please note how it changed?”, and asked for respondent’s age, sex, and country of residence. We used a mixed-methods approach to study birders’ reactions to the COVID-19 pandemic. We carried out qualitative content analyses following the methods of Mayring [43,44]. In such an open-ended qualitative analysis, the researcher is open to the responses of the participants. This is complementary to pure hypothesis testing, because some aspects could not have been considered in advance but were also used for the analyses. Afterwards, we used a mixed methods approach to statistically analyze the data [45]. 

### 3.2. Qualitative Content Analysis

The following classification categories were used: “Did COVID-19 change your birding activity?”: Yes = 1, No = 2, undecided = 3 when two situations were portrayed, e.g., when people live in more than one region or when comparing job versus hobby. The last category was later rejoined with “1 = yes” because some aspects changed, e.g., in the responder’s basic living site but not in her/his summer-cottage site. The second category was whether there was a “reason given”, coded into 1 = yes and 0 = no reason given. For the responses “no”, please see Supplementary Material File S5. The coding of answers in section “yes” is depicted in Table 1.

Most of the coded categories are directly related to our study hypotheses, but some only indirectly (Table 1). For example, reports related to cancelled meetings or increased use of social media can be only indirectly related to changes related to the social changes hypothesis (H5). Therefore, in the coding example Table 1, we highlighted them only as an explanatory part of the study questions. 

### 3.3. Statistical Analysis

We used SPSS 26.0 (IBM, Armonk, NY, USA) to calculate chi-square tests, Spearman’s rank correlation, ANOVAs, and T-tests. We used partial eta-squared as a measure of effect size. Chi-square test was used for the comparison of categories (H1, H2, H3). ANOVAs and T-tests were used to check our hypotheses on gender and age differences (H4). We also used descriptive statistics (frequency percentages were used) and Spearman correlation (H5). For the analysis of gender and age, we used only variables with more than *n* = 200 mentions. Concerning the between-country comparative analysis, we restricted the analysis to countries with at least *n* = 20 respondents. We included all responses (*N*= 4484), but for the analysis of gender and age, data were *n* = 4441 for gender and *n* = 4466 for age, because some respondents did not give their age. We only compared men and women because of the low sample size of people who indicated their gender as “diverse” (not considering themselves as male nor female).

We followed the guidelines of the United Nations [46,47]) to define the sovereignty of a country. We used the human development index (HDI, hereafter) for correlations. The HDI data were extracted from the United Nations Human Developmental Report, where countries are ranked according to their development status (United Nations Development Programme 2018 [46,47]). HDI integrates economic information and measures of human development to obtain an overall score of human development. A high value indicates a high developed country and low value indicates a less developed country. We used the Oxford Coronavirus Government Response Tracker (OxCGRT), hereafter) stringency index (SI, hereafter; [48]) as an indicator of the different countries’ containment and closure policy. The OxCGRT systematically collects information on several different common policy responses that governments have taken to respond to the pandemic on 17 indicators, such as travel restrictions, stay at home requirements, restriction on gatherings, and public info campaigns. The SI is calculated using the policy indicators C1–C8 and H1 (including indicators: c1 schoolclosings, c2 workplaceclosings, c3 cancelpublicevents, c4 restrictionsongatherings, c5 closepublictransport, c6 stayathomerequirements, c7 domestictravel, c8 internationaltravel, and h1 publicinfocampaign). The value of the index on any given day is the average of nine sub-indices pertaining to the individual policy indicators, each taking a value between 0 and 100. The higher the SI, the greater the containment and closure policy in the specific country. In this article, we refer to “*N*” as the sample size, whereas “*n*” refers to the number of cases reported. As this is a qualitative study, *n* refers only to respondents that named a specific reason. Percentages were calculated in relationship to the total sample size (*N*). 

## 4. Results

A total of *N* = 4484 (2834 men, 1607 women, 12 diverse, 31 prefer not to answer) responses were received from 97 countries (Supplementary Material File S4). The mean age was 55.1 years (SD = 16.1). The mean age of males (X = 54.08, SD =16.29) and females (X = 57.02, SD = 15.53) differed significantly, with women being older than men in our sample (t = −5.858, *p* < 0.001). In total, 85% of respondents (*n* = 3802 cases) indicated that COVID-19 changed their birding behavior, and only 15% of respondents (*n* = 682) reported no effect at all. 

Most participants gave reasons and explanations for why their behavior changed or not. People responding with “no changes” in birding due to the pandemic explained less often why their behavior had not changed (Χ^2^ = 2883.46, *p* < 0.0001, df = 1; Supplementary Material File S5). Hereafter, we focus on respondents reporting changes in their birding behavior due to the pandemic (Table 2). In total, 170 people (3.8%) reported that their birding activity has reduced to zero (*N* = 4484). In total, 60% of the respondents (*n* = 2668) reported spatial changes of their birding activity (*N* = 4484; Table 2). 

Birding became more local, and people focused on their nearer environments and birding hotspots closer to their home. During the pandemic, many people (*n* = 1070 cases; 24% of all respondents; *N* = 4484) focused on yard birding, which includes different facets, such as feeder watching, watching from a balcony, rooftop, or within the immediate neighborhood (Table 3). In total, 1.7% of respondents (*n* = 74 cases) reported that they only watched birds from their windows (*n* = 4484), which can be considered as the most restricted form of birding. In total, 0.7% of respondents (*n* = 33 cases) mentioned driving to remote areas for birding and to avoid others, and 0.3% of respondents (*n* = 11) reported visiting and exploring new places and birding hotspots (*n* = 4484). 

### 4.1. Temporal Changes in Birding

In total, 12% of respondents (*n* = 542 cases) reported having more time for birding, and 8% (*n* = 356) reported less time for birding (*N* = 4484), which were statistically significant (binomial test, *p* < 0.001). About 20% (*n* = 898 cases) of the respondents indicated some temporal changes in their birding behavior, including circadian changes (*n* = 41 cases; see Table 2). Circadian changes were mostly shifts toward earlier birding times in the morning (*n* = 26 cases) or later in the evenings (*n* = 1), while some avoided “crowded times” (*n* = 11) or preferred bad weather with rain (*n* = 3).

### 4.2. Social Aspects in Birding

Social aspects of the COVID-19 pandemic were reported in a variety of cases (Table 2). The most important change was the shift to no group birding (or with spouse) but also cancellations of field trips, events, and group outings with a bird club were often reported. In addition, carpooling was reduced, as was equipment sharing, while people focused more on keeping distance from others (Table 2). Concerning social media, 1.3% of respondents (*n* = 59 cases) explicitly mentioned eBird as a tool that helped structuring the reporting or in finding closer less crowded hotspots. In total, 2.3% of respondents (*n* = 102 cases) reported more digital web activity, including online courses, Facebook, and listserv amongst others. 

### 4.3. Change of Birding Activities and Content

COVID-19 also had a severe impact on monitoring programs and breeding bird surveys. Respondents reported cancellations in monitoring schemes or surveys, or in ringing/banding, and cancelations in bird tours and walks that they were supposed to lead or where they participate (Table 2). Additionally, the main content of birding activities changed throughout the crisis, with a stronger focus on bird behavior, improving one’s identification skills, listening to nocturnal migration, and others (Supplementary Material Table S6), while the chasing of species (twitching) or the listing became less important. 

### 4.4. Role of Age 

We assessed whether answer categories differed in age. No difference in age existed in the answer to the general question if COVID-19 had changed birding behavior (Table 3). However, there were significant age differences in the variables increased solo birding or with spouse, field trips cancelled, and holidays cancelled. In all three variables, participants were older (Table 4). There was no difference concerning time for birding (T = −1.24, df = 895, *p* = 0.217) and in the different categories of spatial change (ANOVA F = 1.45, df = 4, *p* = 0.214), and the aspect of cancelled surveys and monitoring plans (Table 4). 

### 4.5. Role of Gender

Men reported more often than women that COVID-19 did not change their birding activity (χ^2^= 6.02, df = 1, *p*= 0.014). There were no statistically significant differences between men and women in the categories concerning spatial changes (χ^2^ =7.40, df = 4, *p* = 0.116), in holidays cancelled (χ^2^ = 0.018, df = 1, *p* = 0.893), and in surveys cancelled (χ^2^ = 2.63, df = 3, *p* = 0.453). However, there were significant differences in temporal changes (χ^2^ = 19.49, df = 2, *p* < 0.001), avoidance of group birding (χ^2^ = 20.51, df = 1, *p* < 0.001), and in field trips cancelled (χ^2^ = 26.69, df = 1, *p* < 0.001).

### 4.6. Between-Country Comparison

**Figure 1:**
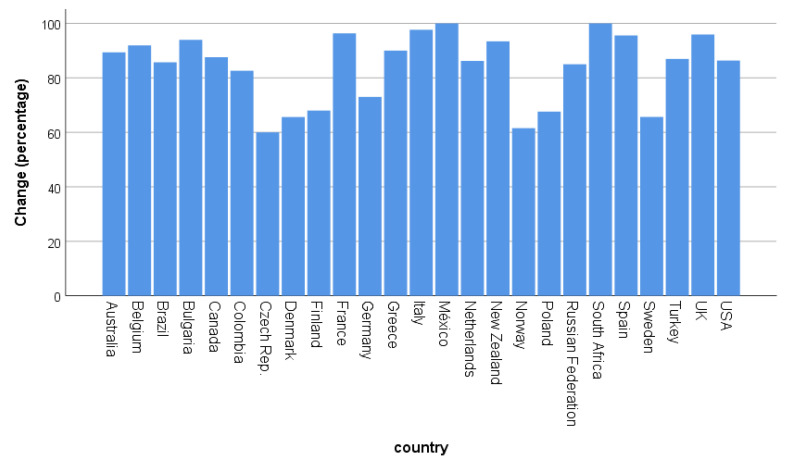
Percentage of people responding with “Yes” to the question “Did COVID-19 change your birding behavior?” according to country. Country-based N is given in the Supplementary Material File S4.

We found a general difference between countries concerning the percentage of people reporting that COVID-19 changed their birding behavior (χ^2^ = 290.08, df = 24, *p* < 0.001, Figure 1). The lowest percentages (68% and lower) were reported in Czech, Denmark, Finland, Norway, Poland, and Sweden while 96%-100% of the participants reported changes from France, Italy, Mexico, South Africa, Spain, and the UK.

**Figure 2:**
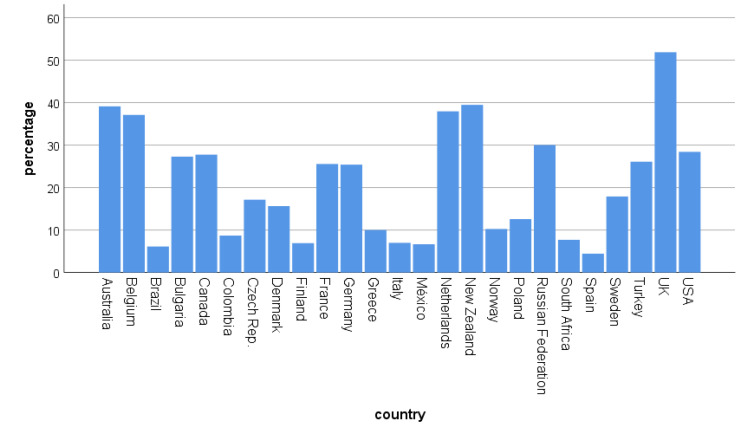
Spatial changes (more yard birding; closer to home) of birding according to country (percentage of people). Country-based N is given in the Supplementary Material File S4.

**Figure 3:**
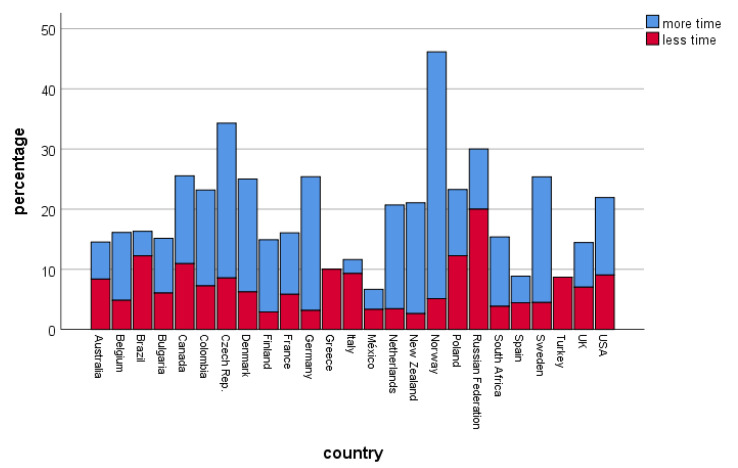
Temporal changes among countries (percentage of people). “More time” refers to people having more time for birding, while “less time” refers to less time spent for birdwatching during the COVID-19 pandemic. Country-based N is given in the Supplementary Material File S4.

Spatial changes in birding behavior also differed between countries (χ^2^ = 302.41, df = 24, *p* < 0.001, Figure 2). In some countries (Colombia, France, Greece, Italy, Mexico, South Africa, and Spain), a shift toward yard birding occurred, which means a strong reduction in visited places and a restricted spatial scale of birding. While in other countries spatial changes were mostly related to birding closer to home, e.g., Czech Republic, Denmark, Finland, Norway, Poland, and Sweden. Those were also the countries where people mentioned being less affected in their birding behavior by COVID-19 (see Figure 2). There were also differences in changes of temporal activities (χ^2^ = 138.08, df = 48, *p* < 0.001, Figure 3). In some countries, time spent birding increased, while it decreased in others. However, on average, people reported spending more time for birding than less time for birding.

**Figure 4:**
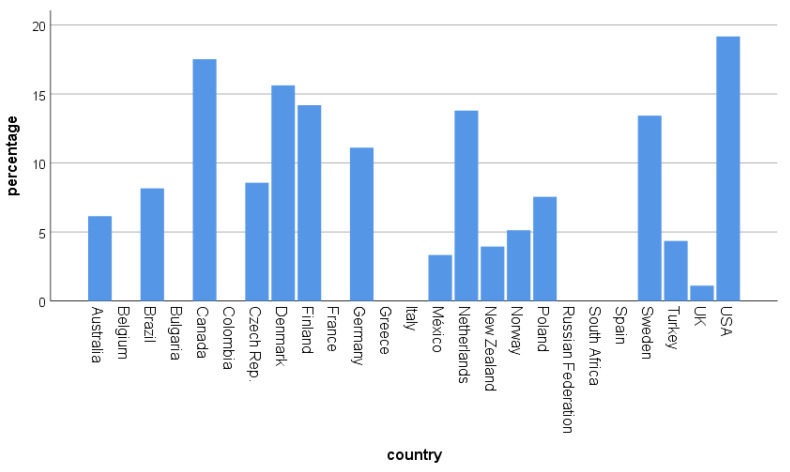
Percentage of people mentioning the avoidance of group birding (birding only alone or with spouse/family member) according to countries. Country-based N is given in the Supplementary Material File S4.

The change toward avoidance of groups differed between the countries (χ^2^ = 234.04, df = 24, *p* < 0.001; Figure 4). A transformation to avoiding group birding was primarily found in Canada, Denmark, Finland, Germany, the Netherlands, Sweden, and the US. In a correlational approach, we related the answers (percentage per country) with the HDI (Table 5). Higher HDI, and thus higher development, was positively correlated with spending more time birding, more group avoidance, more fieldtrips cancelled, more holidays cancelled, and with birding closer to home (Table 5). Similar results were obtained when the SI was used.

## 5. Discussion

This study focused on an outdoor leisure activity and the relationship to the pandemic. There seem to be no studies about this topic yet, and we believe that the results are somewhat generalizable to other nature-related outdoor-leisure activities, such as angling/fishing or hunting. We found a strong dynamic in behavioral responses, i.e., spatial, temporal, circadian, and social effects, toward birding activities during the COVID-19 pandemic. These effects also differed between countries. 

Our results indicated that COVID-19 influences the behavior of birders—either directly or indirectly via containment and closure policy. People reported more often that their birding behavior was affected by COVID-19. This was an expected result, because previous work showed a significant influence of the pandemic and governmental measures on human behavior [2,3,4,30,35]. It is interesting that people also reasoned why their birding behavior had not changed. In some cases, it is intuitive, because birders avoiding group birding may not experience the social distancing as a restriction. Additionally, people living in the countryside with a good patch for birdwatching may not experience travel restrictions because they did not travel previously. 

Hypothesis 1 (spatial changes) could be only partially confirmed. Spatial effects were found in most countries with increasing birding within yards or near surroundings. Obvious reasons were the governmental orders, travel restrictions, and stay at home orders or guidelines for elderly (>70 years, e.g. in Finland) because COVID-19 can be harmful especially for them [49]. Our results also indicated that birders try to find new, not overcrowded birding sites. By doing so, possibly new high-quality birding sites can be found. The changes in birding behavior due to the pandemic resulted in an increase in the utilization of green spaces in cities [35]. 

Hypothesis 2 (temporal effects) could be only partially confirmed. Temporal effects are two-fold, some people pursue birding activities more (e.g., because of working from home/ having more time/ no commuting times), others less (e.g., due to the travel restrictions). Some responders indicated that they go birding more often on weekdays during the COVID-19 pandemic than before, probably because they now have more time during the week and want to avoid weekend rushes in the most popular birding sites. If this is true in a larger content, the COVID-19 pandemic will increase the data quality of citizen science-based bird monitoring projects by decreasing the weekend bias of the data quality and quantity (because usually, many birders go birding during the weekend [50]). 

However, some Finnish respondents stated that due to the local travel restrictions, they were unable to conduct their voluntary bird monitoring duties. We also found some circadian shifts in birding behavior. Some birders (26 out of 898; about 2%) reported going birding earlier than before COVID-19. By doing so, they tried to avoid rush hours, because otherwise they must queue to be able to visit a bird tower. The change in circadian rhythms can also influence the data collection, amount, and quality since bird activity is usually high at sunrise (dawn chorus), and this may lead to an accurate assessment of species richness and breeding pair numbers. However, more data are needed to generalize the effects of COVID-19 for birders’ circadian behaviors.

Hypothesis 3 dealt with the social behaviors of birders. Many birders reported that public bird happenings were cancelled due to the pandemic. In addition, birders preferred to go birding with their friends within a small group. Due to COVID-19, they did not share their car with others other than their own family members. Our results clearly indicated that solo birding or birding with spouse increased heavily during the COVID-19 pandemic. Birding is a social activity. Gaining respect from other birders, building friendships, and meeting people that share the same interests are important motivational factors for birding [13]. Therefore, the COVID-19 pandemic may have a negative influence on the well-being and social interactions of birders.

Concerning hypotheses 4 (demographic aspects), COVID-19 was related to age and gender. Older people seemed to be more affected than younger ones by decreased group birding, cancelled field trips, and holidays too. We can only speculate about this, but older people may feel less safe when birding alone (e.g., fear of crime [51]) and are more at risk of infections [28]. Women more than men seem to be birders that need social contact and suffer from social distancing because they more often complained about cancellations of field trips or organizational meetings. Further, they mentioned increased group avoidance during the COVID-19 pandemic more often. In line with this, when analyzing the initial involvement in birding, women more often started birding by participating in an organized walk or a birding club activity [16,22], suggesting that the social aspects of birding may have a stronger influence on women, which matches our results concerning the affection of cancelled trips and club activities. 

With regard to hypothesis 5, we found a strong correlation between the HDI and our dependent variables. This indicates that the experienced changes in lockdown are related to economic development. Cancelled trips/holidays were mentioned more often by people from countries with a higher HDI [16,17]. Additionally, in higher developed countries, spatial travel restrictions were mentioned more often by the respondents. This may be the case because travelling to farther birding hotspots is more common than in countries with a lower HDI. In those countries, birding trips are more likely to take place nearby [52]. Concerning temporal changes, people in countries with a high HDI experienced the effect of having more time, because their work was probably shifted toward remote working at home. Field trips with birding clubs and group birding seem to be more affected in countries with high HDI. These may be more common in those countries, and therefore, the loss of those activities can be experienced more [33]. The results concerning the stringency index (SI) were similar, with a higher stringency showing a stronger response.

One limitation of our work is that it was a snapshot short-questionnaire study. It was intended to address a high number of respondents. In general, our study population represents the birding community well. Most respondents were middle-aged men. Other variables should have been added, such as the current work, living habitat (e.g., urban vs. rural), years of carrying out the birding activity, or some recreation specialization questions. However, recreational specialization levels of birders and demographic factors (e.g., gender) might be interrelated. Our results indicated that women reported more often than men about cancellations of society meetings. This can also be related to gender differences in the specialization level. However, a pandemic is an unpredictable phenomenon. Thus, it is crucial for research to react quickly and start surveys soon, because people do not remember facts correctly afterwards, which is known as hindsight bias in psychology [53]. We, therefore, believe that our study is valuable in terms of data collection during the pandemic. In addition, we used Excel and SPSS directly for coding the answers, and not another software, for qualitative analyses. This could be viewed as another limitation.

## 6. Implications

There are many implications of this study. First, it shows that governmental decisions are always a trade-off between the highest possible protection from an infection and keeping life ongoing. Long lockdowns with strict regulations may severely impact leisure activities that are part of human life. This will influence, e.g., the physical and mental health of people. Additionally, social distancing or birding alone was mentioned often, indicating a negative influence on human social life and well-being. In practice, birders and other leisure activists can change their circadian pattern to avoid crowding temporally. Concerning spatial changes, sites near home may experience a higher load of visitors, which can have management implications. Further, a temporal and spatial shift in birding may influence the data quality in citizen science projects. 

## 7. Conclusions

To our knowledge, this is the first study where interactions between large-scale pandemic and behavioral changes in a nature-based leisure activity (birding) were assessed. We conclude that the COVID-19 pandemic has severe impacts on birding content, birders’ behavior, and social interactions as well as their contribution to citizen science projects. Nature-based recreation will be directed more toward nearby sites in the neighborhood; therefore, environmental management resources and actions need to be directed to sites that are located near the users, e.g., in urban and suburban areas. 

## Figures and Tables

**Table 1:** A general overview of the relationships of hypotheses and coding of responses.

Hypothesis	Category	Coding; Variables Used
H1	Spatial changes in behaviors	Spatial changes of birding were coded when responder indicated that: birding: *-was restricted or limited on fewer sites or sites changed during the COVID-19 pandemic/lockdown* *-was more concentrated in yard;* *-was performed closer to home or birding was more local;* *-was performed in under-birded, less popular, less crowded sites;* *-was more often directed on early unknown or less-visited birding sites*
H2	Temporal changes in behaviors	Temporal changes of birding were coded when responder indicated that: *-has either more or less time for birding during the COVID-19 pandemic than earlier;* *-performed birding either earlier or later during the COVID-19 pandemic than before;* *-avoided birding during crowded times*
H3	Changes in Social behaviors	Social changes in birding behaviors were coded when responder indicated that: *-was forced to bird alone or with his/her spouse or partner only* *-birding with friends (other than spouse or partner) was impossible;* *-club meetings, festivals, bird-related holidays, tour guide/group walk leader/workshops were cancelled;* *-bird survey/monitoring stopped, bird ringing/banding stopped, tour guide/group walk leader/workshops cancelled* *-social media use, like Facebook, eBird, virtual birding, social media, SMS, online tools was higher during the COVID-19 pandemic than before;*
H4	Role of demography factors	Age was coded as years; Gender was coded as man or woman
H5	Country differences in behaviors	Human Development Index (HDI, values 0–1); Stringency Index (SI, values 0–100)

*Coding example:* “I stay “local, not traveling” more than five miles. I bird in “remote locations” and only “alone”. I also bird a lot in my “suburban neighborhood”, walking a 1–2 mile loop. I go “early in the morning” before most neighbors are up and about, and those that are do respect physical distancing. A mega rarity was found two days ago in my state and I chose “not to chase” because we are on a stay-at-home order. […]“. This was coded into: (1) Yes, COVID-19 changed behavior, (2) Yes, reason given, (3) Spatial: closer to home, (4) No group birding, (5) circadian effect: going earlier, (6) no twitching. So, six categories could be coded from this answer.

**Table 2:** Overview of the changes in birding behavior during the COVID-19 pandemic. Percentage is given in relation to the full sample of *N* = 4484. Percentages can exceed 100% because participants could select more than one response.

Outcome/Reason	*n* Cases	Percentage
Changes Specifically Related to Birdwatching		
Spatial change	2668	59.5%
Temporal change	898	20%
Changes due to no group birding	531	11.8%
Circadian change	41	0.9%
Explicitly visiting less rewarding places	92	2.1%
No or less twitching	70 *	1.6%
Changes not specifically related to birdwatching		
No carpooling	51	1.1%
Keeping distance	45	1.0%
Avoiding (overcrowded) towers/hides	42	0.9%
No equipment sharing	21	0.5%
Field trips, meetings, cancelled	363	8.1%
Holidays, international travel cancelled	301	6.7%
Surveys cancelled	235	5.2
Avoiding crowds	17	0.4%

* one person reported even more twitching activity; a twitcher is considered a birder who responds with frenzied activity to news of rarities in his/her region, and will spend money and travel long distances to see a rarity or new bird [16].

**Table 3:** Spatial changes in birding during the COVID-19 pandemic. Percentage is given in relation to the full sample of *N* = 4484. Percentages can exceed 100% because participants could select more than one response.

	*n* (Cases)	Percentage
More yard birding	1070	23.9
More birding in closer environment and local areas	1104	24.6
Less birding at remote and over-used areas	229	5.1
Birding is more restricted or limited	265	5.9
Total	2668	59.5

**Table 4:** Age comparison of different activities. A response indicating “No” meant that the respondent did not mention this in the open-ended question. “Yes” means it was explicitly mentioned. For a general non-age-stratified analysis, see Table 1 and Table 2. SD is standard deviation.

	No		Yes		F	*p*	Eta-Squared
	Age	SD	Age	SD			
Did COVID-19 change birding?	55.88	16.26	54.96	16.09	1.86	0.173	0.000
Field trips cancelled	54.55	16.17	61.36	14.15	60.27	<0.001	0.013
Avoid group birding	54.43	16.17	60.07	14.83	57.84	<0.001	0.013
Holiday cancelled	54.94	16.23	57.41	14.43	6.59	0.01	0.001
Survey/monitoring cancelled	55.13	16.17	54.69	15.34	0.16	0.686	0.000

**Table 5:** Relationship between HDI (human development index) and SI (stringency index) per country and percentage of changes reported. Spearman’s rank correlation.

Reported Changes		HDI	*p*	Bonferroni Adjusted	SI	*p*
Changes in birding activity	r_s_	−0.362	0.075	ns	0.388	0.056
Temporal change (more time)	r_s_	0.539 **	0.005	‡	−0.192	0.359
Group avoidance birding	r_s_	0.573 **	0.003	‡	−0.607	0.001 ‡
Surveys cancelled	r_s_	−0.162	0.44	ns	0.229	0.272
Fieldtrips and club activities cancelled	r_s_	0.510 **	0.009	‡	−0.547	0.005 ‡
Holidays cancelled	r_s_	0.604 **	0.001	‡	−0.467	0.019
Spatial: more yard birding	r_s_	−0.684 **	<0.001	‡	0.457	0.022
	P					
Spatial: closer to home birding	r_s_	0.438 *	0.028	ns	0.174	0.405
	P					

** indicates *p* < 0.01; * indicates *p* < 0.05; ‡ indicates significant after Bonferroni correction (*p* = 0.006). ns = not significant. Bonferroni was applied with 0.05/8 = 0.006.

## Data Availability

The data that support the findings of this study are available from the corresponding author upon reasonable request.

## Supplementary Materials

The following are available online at https://www.mdpi.com/1660-4601/17/19/7310/s1, File S1: Recruitment e-Mail, File S2: Posting on a website, File S3: Posting on Facebook, File S4: Overview over the responses per country, File S5: Reasons when COVID-19 did not change the behavior of birdwatchers, File S6: Change of birdwatching content/activities during the COVID-19 outbreak.
