# Supplementary material for: SARS-CoV2 (COVID-19) Pandemic Lockdown Influences Nature-Based Recreational Activity: The Case of Birders
Source: Int J Environ Res Public Health. 2020 Oct 7;17(19):7310. doi: 10.3390/ijerph17197310 (PMC7579058; doi:10.3390/ijerph17197310)
Supplement: Supplementary file 1 [file ijerph-17-07310-s001.pdf]

International Journal of Environmental Research and Public Health

MDPI

Supplementary Material 1

Recruitment e-Mail

Dear fellow birdwatchers,

Corona has changed our lives and we want to study its influence on birdwatching activities in different countries. Please help us and answer a few simple questions. The questionnaire is in seven different languages and takes 2 (two) minutes. The study is run by Piotr Tryjanowski (University of Poznan) and Christoph Randler (University of Tuebingen).

https://www.soscisurvey.de/birds_corona/

Stay healthy,

Christoph & Piotr

More information:

It is a scientific study, and you can check my profiles on the website of the University of Tübingen/MNF, Researchgate, eBird, Facebook (BioDidaktik Tübingen), etc.; I am a birder myself, working at the intersection of biology & social sciences, so thank you all for helping us getting a clearer picture how corona impacts on social/psychological aspects of a leisure activity.

Please take care and stay healthy,

Christoph

Supplementary Material 2a. Posting on a website.

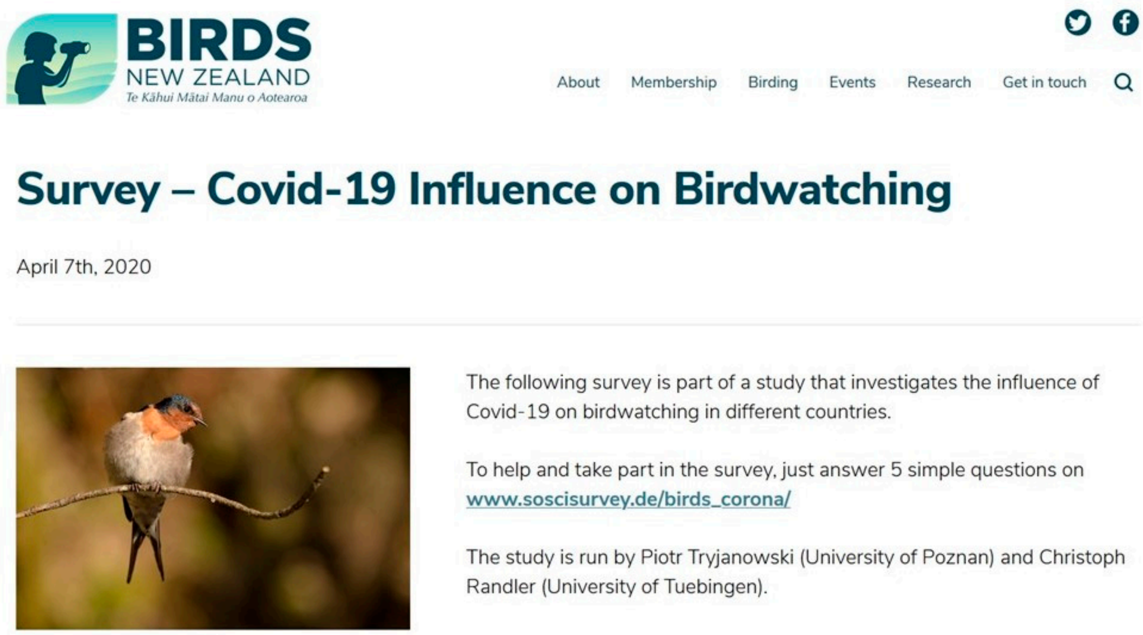

Supplementary Material 2b.

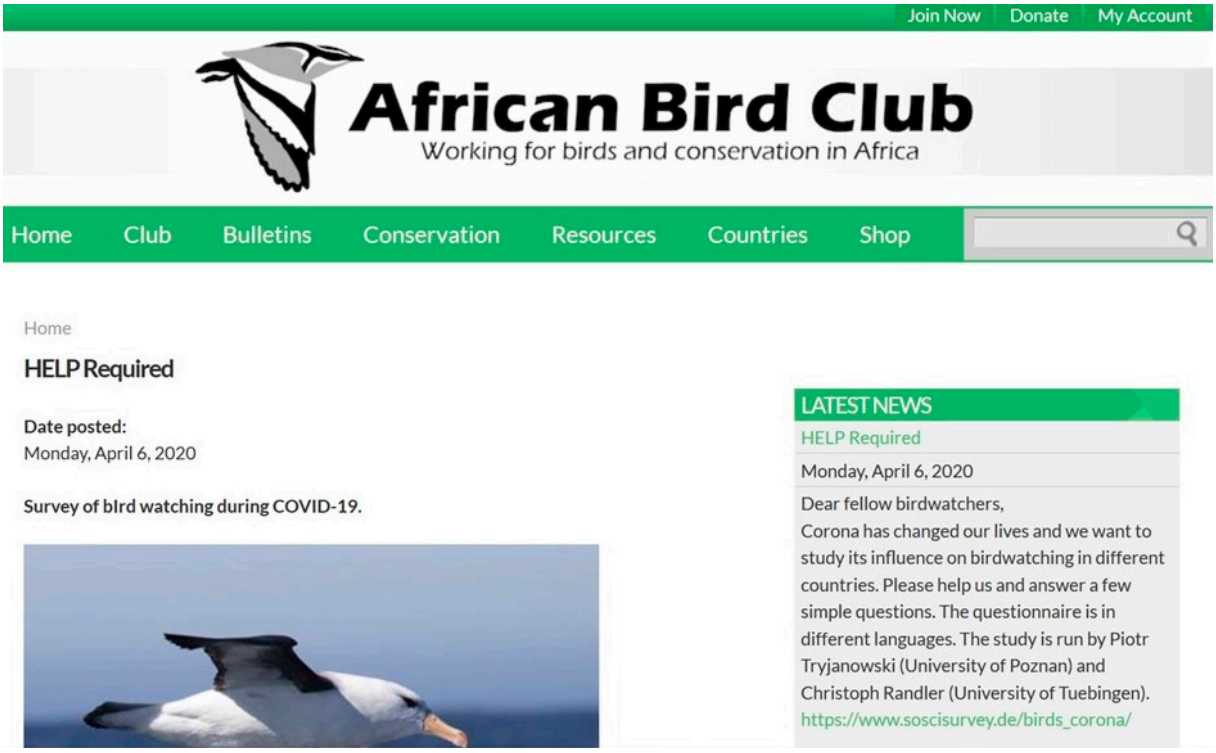

Supplementary Material 3. Posting on Facebook

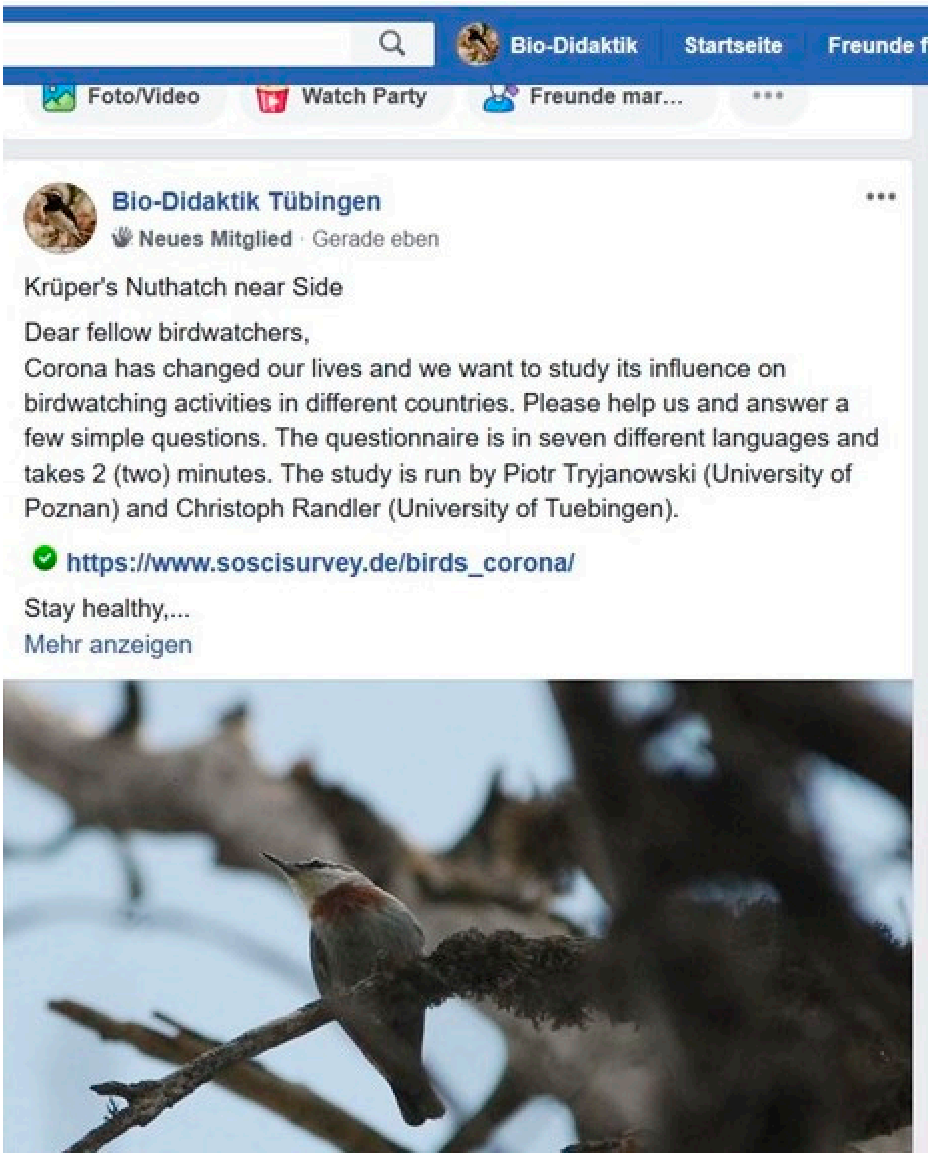

Supplementary Material 4. Overview over the responses per country.

country	N	Percent
Albania	1	0
Algeria	7	0.2
Argentina	4	0.1
Armenia	1	0
Australia	179	4
Austria	7	0.2
Bahamas	1	0
Bahrain	1	0
Bangladesh	2	0
Belarus	2	0
Belgium	62	1.4
Belize	4	0.1
Bhutan	1	0
Bolivia	2	0
Brazil	49	1.1
Bulgaria	33	0.7
Canada	137	3.1
Chile	4	0.1
China	4	0.1
Colombia	69	1.5
Congo Brazzaville	1	0
Costa Rica	4	0.1
Croatia	2	0
Cuba	1	0
Cyprus	12	0.3
Czech Rep.	35	0.8
Denmark	32	0.7
Dominican Republic	1	0
DR Congo	1	0
Ecuador	3	0.1
Egypt	8	0.2
Estonia	14	0.3
Ethiopia	1	0
Finland	275	6.1
France	137	3.1
Gambia	1	0
Germany	63	1.4
Ghana	2	0
Greece	20	0.4
Guatemala	1	0

Hungary	9	0.2
Iceland	1	0
India	6	0.1
Iraq	1	0
Ireland	7	0.2
Israel	9	0.2
Italy	86	1.9
Japan	14	0.3
Jordan	1	0
Kazakhstan	1	0
Kenya	13	0.3
Lebanon	1	0
Lithuania	2	0
Luxembourg	12	0.3
Malawi	1	0
Malaysia	2	0
Malta	6	0.1
Mauritius	1	0
México	30	0.7
Mongolia	2	0
Morocco	1	0
Myanmar	3	0.1
Namibia	1	0
Nepal	1	0
Netherlands	29	0.6
New Zealand	76	1.7
Norway	39	0.9
Panama	3	0.1
Peru	2	0
Philippines	3	0.1
Poland	318	7.1
Portugal	10	0.2
Qatar	3	0.1
Republic of Korea	1	0
Romania	6	0.1
Russian Federation	20	0.4
Saudi Arabia	3	0.1
Serbia	2	0
Singapore	12	0.3
Slovakia	6	0.1
Slovenia	8	0.2
Solomon Islands	1	0

South Africa	26	0.6
Spain	158	3.5
Sri Lanka	12	0.3
Sweden	67	1.5
Switzerland	12	0.3
Trinidad and Tobago	2	0
Turkey	23	0.5
UAE	2	0
Uganda	4	0.1
UK	271	6
UR Tanzania	8	0.2
USA	1957	43.6
Venezuela	3	0.1
Viet Nam	1	0
Zambia	1	0

Supplementary Material 5. Reasons when COVID-19 did not change the behavior of birdwatchers If COVID-19 did not change the behavior of a respondent, some still reported reasons for this. . *These answers were coded in a separate section "No"*: We grouped respondents to the category "yard" when their birding behavior took place exclusively from home before and during the pandemic (birding from windows, backyard, feeder, or on own property). We coded "uncrowded place" when birding before COVID-19 already occurred in spots without many people, in under birded areas, in rural/remote areas, in a time frame outside usual birding times, or when avoiding crowds or crowded places by spatial/temporal avoidance behavior was mentioned and therefore did not change. We coded "no group" when birding before COVID-19 already occurred only alone or in small groups of two people (mostly with a spouse/partner or family member; because grouping was prohibited but with spouse/partner was allowed by governmental restrictions). We coded "local spot" when birding before COVID-19 already occurred near home, but outside one's own property. "Season" was coded when the season was too early for birding activities (e.g. in Alaska). "Car" was coded when respondents' birding activities take place in a car. "No time" was assigned when the participant had no time for birding before and during the pandemic; "bird job" was used when birding was part of the respondent's job and was not yet suspended.

Focusing on the respondents without experiencing any changes, most of them avoided crowded places, are yard, car or group avoidance birdwatchers or have a local spot already prior to the COVID-19 outbreak (SFigure 1). Some of them were professionals being able to continue their field work, or mentioned they had no time for birding anyway or that the season had not started yet. Therefore, the pandemic had no significant influence upon their behavior.

Figure 1. Reasons when COVID-19 did not change the behavior of birdwatchers.

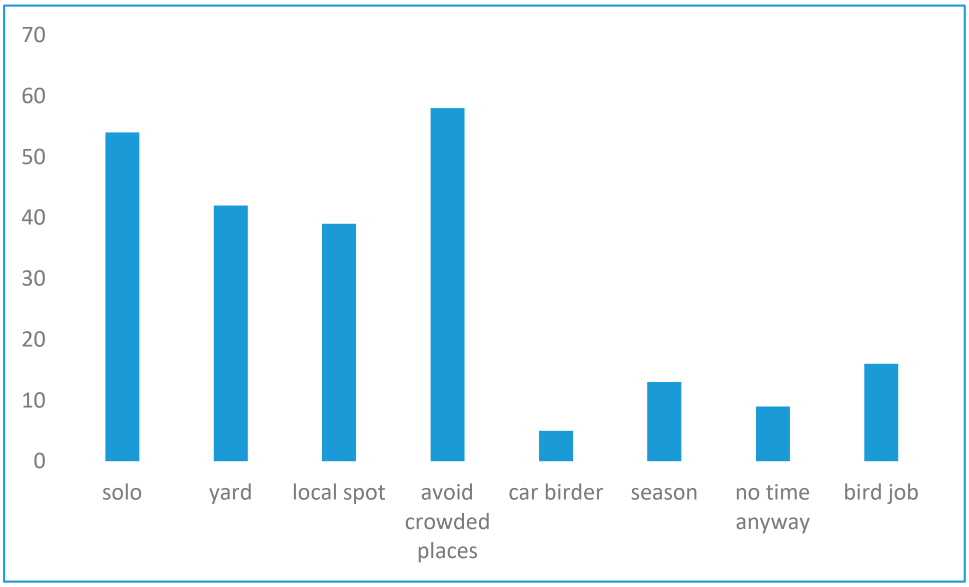

Supplementary Material 6. Change of birdwatching content/activities during the COVID-19 outbreak.

Activity	N	percentage
More attention on bird behavior	13	0.3
Develop identification skills	4	0.1
Making now gardenlist	11	0.2
More listening to birds	8	0.2
More observation of the nocturnal migration	8	0.2
More attraction on the Phenology (arrival dates)	2	0
Rediscover common avifauna (appreciation)	9	0.2
